# When cold adjusts volatile metabolism: CBF4 and WRKY4 activator-repressor module controls nerolidol glucosylation in tea plants

**DOI:** 10.1093/plphys/kiag083

**Published:** 2026-02-25

**Authors:** Eva Maria Gómez-Álvarez

**Affiliations:** Assistant Features Editor, Plant Physiology, American Society of Plant Biologists; Institute of Plant Sciences, Scuola Superiore Sant’Anna, Pisa 56010, Italy

Plants continuously adjust the amount, activity, and distribution of small molecules to survive abiotic stresses, such as low temperatures. As an example, metabolite glycosylation, which affects plants’ activity and localization, contributes to their responses to a changing environment by modulating the activity and localization of selected compounds (Wang et al. 2019). Glycosylation is mediated by UDP-dependent glycosyltransferases (UGTs), enzymes that conjugate sugars to small molecules, including hormones, volatiles, or secondary metabolites (Poppenberger et al. 2005; Liao et al. 2023). As an example, Zhao et al. (2022) demonstrated that glycosylation of abscisic acid (ABA) in *Camellia sinensis* (tea plants) enhances cold and drought tolerance. Thus, UGT-mediated glycosylation plays crucial roles in mediating plant responses to biotic and abiotic stresses.

Volatile organic compounds (VOCs), like sesquiterpenes, are important components of plant stress responses, acting both as protective metabolites and as signaling molecules (Jin et al. 2023). Nerolidol is a sesquiterpene that is distributed across plant species and functions as a volatile signal perceived across different trophic levels. Nerolidol displays a phytohormone-like activity, enhancing plant defense through MAPK signaling, WRKY transcriptional regulation, jasmonic acid, and ABA dependent pathways (Green et al. 2011; Chen et al. 2020). In tea plants, nerolidol glucosylation a specific form of metabolite glycosylation, is catalyzed by CsUGT91Q2, a cold-induced UGT that contributes to cold acclimation. However, how cold perception is linked to UGT activation remains unresolved (Zhao et al. 2020).

Cold stress signaling in plants is normally framed around the CBF/DREB1 cascade (Shi et al. 2017). Although studies in tea plants (Zhao et al. 2020; 2022) have implicated CBFs in coordinating VOC responses to cold, it is still unclear whether these cascades control VOC biosynthesis and modification. The function of WRKY and bHLH transcription factors (Yu et al. 2023) suggests the existence of regulatory modules that may converge on VOC pathways. How these transcription factor networks are integrated to link cold perception with volatile-mediated stress adaptation remains an open and timely question.

In a recent paper in *Plant Physiology*, Yu et al. (2026) identified CsAIF3 (ATBS1-INTERACTING FACTOR), a cold-inducible bHLH transcription factor, as a key regulator of the glycosyltransferase CsUGT91Q2, and described a transcriptional module that links the response to cold stress and to nerolidol modification in tea plants.

Previous transcriptional profiling studies reported a correlation between the expression of CsCBF1–6 and CsUGT91Q2 under cold stress (Zhao et al. 2022). However, Yu et al. showed that none of the CsCBFs directly bind the CsUGT91Q2 promoter. To identify regulators of CsUGT91Q2, the authors constructed a single-cell transcriptome–derived coexpression network linking transcription factors to CsUGT91Q2, which revealed that CsAIF3 directly binds the CsUGT91Q2 promoter and activates its expression.

Functional analyses further supported a role for CsAIF3 in cold tolerance. When exposed to low temperatures, CsAIF3-silenced tea plants displayed significantly reduced maximum quantum efficiency of PSII photochemistry (*F_v_/F_m_*) compared with control plants, consistent with increased sensitivity to cold stress. By combining multiple independent molecular approaches, such as ChIP-Seq and Y2H assays, the authors describe a regulatory mechanism in which CsCBF4 activates CsAIF3, CsWRKY4 acts as a repressive brake on CsAIF3 activity, and cold-induced CsCBF5 relieves the repression to enable CsUGT91Q2 activation.

All together, these findings can be integrated into a model (Fig. 1). Briefly, under non-stress conditions, CsAIF3 is bound by CsWRKY4, repressing CsUGT91Q2 expression and limiting nerolidol glycosylation But, under low temperatures, CsCBF4 directly activates CsAIF3 expression leading to the induction of CsUGT91Q2 and the accumulation of glucosylated nerolidol. Cold exposure relieves the CsUGT91Q2 repression through the action of another transcription factor, CsCBF5, which competitively disrupts the CsWRKY4–CsAIF3 complex, freeing CsAIF3 to activate CsUGT91Q2. Together, this regulatory switch enables temperature-dependent control of nerolidol glycosylation, priming tea plants for cold stress.

**Figure 1:**
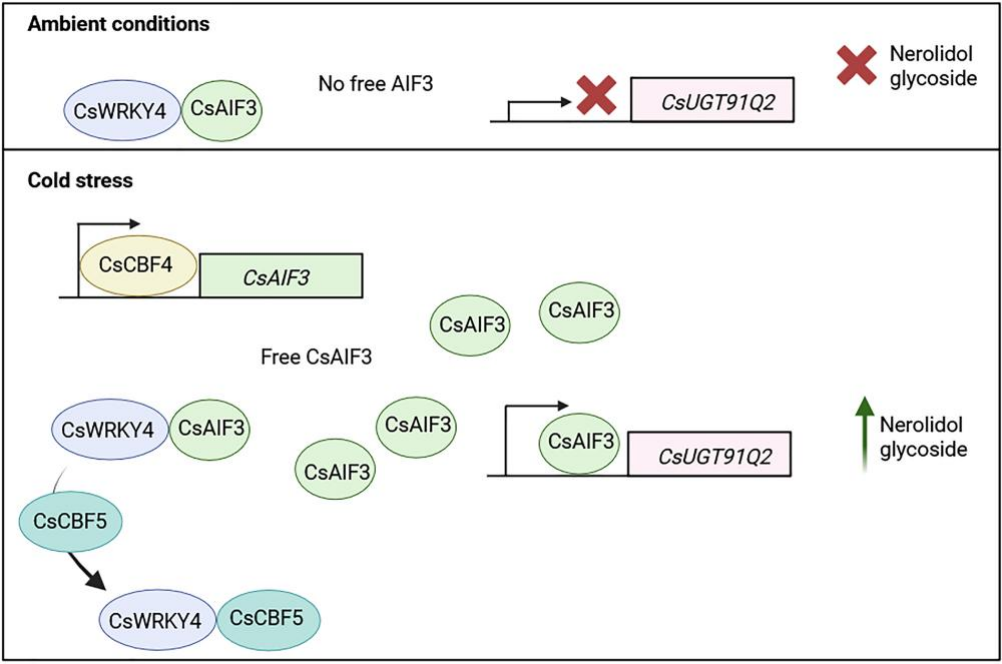
Model based on the findings from Yu et al. 2026. In ambient conditions, CsWRKY4 is bound to CsAIF3, preventing CsAIF3 from activating expression of CsUGT91Q2 and thus avoiding the production of nerolidol glycoside. When cold is perceived, CsCBF4 induces the expression of CsAIF3. Free CsAIF3 binds to the promoter of *CsUGT91Q2* inducing its expression and thus producing the glycosylation of nerolidol. Additionally, in cold conditions CsCBF5 competitively displaces the complex between CsWRKY4 and CsAIF3, further allowing free CsAIF3 to induce expression of CsUGT91Q2 and produce nerolidol glycoside. Figure created with BioRender.

Cold stress represents a challenge for plant growth, requiring fast metabolic adjustment to maintain cellular function. Yu et al. show that volatile modification plays a role in this response, identifying a transcriptional module that links cold perception to the accumulation of a glycosylated nerolidol in tea plants. Their work shows that there is a balance between activating and repressing transcriptional inputs in response to cold stress, enabling the induction of a glycosyltransferase. This switch integrates canonical cold-response pathways with volatile metabolism, allowing plants to tailor metabolite availability when temperatures drop. By connecting transcriptional control with nerolidol modification, the study highlights how plants coordinate stress and metabolic plasticity during cold adaptation.

## Data Availability

No new data were generated or analyzed in support of this research.
